# Develop Your Case! How Controversial Cases, Subcases, and Moderated Cases Can Guide You Through Mixed Methods Data Analysis

**DOI:** 10.3389/fpsyg.2019.01369

**Published:** 2019-06-21

**Authors:** Judith Schoonenboom

**Affiliations:** Department of Education, University of Vienna, Vienna, Austria

**Keywords:** mixed methods, data analysis, educational research, case development, controversial case, subcase, moderated case

## Abstract

In this article, we present mixed methods data analysis in educational research as a process of *case development*. We define a case as a theoretical construct that is developed through iterative and spiraling cycles of interaction between ideas and evidence. On a larger scale, cases develop over a period of several years or even decades, involving many studies. But cases also develop within a much smaller timescale, within studies. Development of cases within mixed methods studies is the theme of this article. We define the purpose for any mixed methods study as to *acquire detailed insight into the case, subcases, and the way the subcases are moderated.* Developing a case starts with identifying a phenomenon that will be the object of research. A phenomenon can be investigated empirically once researchers are able to identify specific contexts, localized in time and space, in which the phenomenon occurs. At that point, an *underdeveloped case* has come into being, and the specific contexts in which the case occurs are called *instantiations* of the case. Connected to each case are *claims*, statements about the case that are used to describe the case more precisely and to distinguish cases from non-cases. Developing these claims is the aim of the analysis processes. Case development includes three different research processes—namely, resolving a controversial case, developing subcases, and developing a moderated case. Case development typically occurs in this order; however, each of these processes *can* but do not *need* to be present. When cases develop, they typically become *controversial* for some time. Some research studies support a specific claim, while other research studies refute this claim. Next to claims that apply to all instantiations of a case, claims may be developed that apply to only some of the case’s instantiations. In that case, claims are used to distinguish *subcases*. A *moderated case* is developed when researchers come to understand under which circumstances their claim does and does not apply, which leads to their subcases. This is called “moderation” of their claim.

## Introduction

This article examines data analysis in mixed methods educational research, henceforth referred to as *mixed analysis*. Mixed analysis presents a challenge for mixed methods researchers from the very beginning of an inquiry until the very end. The challenge begins with research design. At the beginning of a study, the researcher must develop a plan that describes various aspects of mixed analysis. These aspects include how the results of separate quantitative and qualitative analyses will be amassed, how qualitative and quantitative data are brought together during analysis, and how qualitative and quantitative data collection and data analysis proceed together. Typically, not all aspects of a mixed analysis can be planned before the study starts. Thus, in addition to prior planning, the researcher will often have to engage in unforeseen forms of mixed analysis during the research process. Another challenge that especially affects evaluation research and case study research is the sheer amount of qualitative and quantitative data that become available in the course of the study and the challenge of what to analyze and how to analyze these various data types together.

Thus, a question arises in relation to what principles can guide researchers through the challenging process of mixed data analysis. One fruitful approach to mixed analysis assumes that the qualitative and quantitative data engage in a *dialogue*. The idea of research as a form of dialogue is not unique to mixed methods research. Within case development, for example, “[i]nteraction between ideas and evidence results in a progressive refinement of the case conceived as a theoretical construct” (Ragin, 1992, p. 9). Mixed methods scholars have added that this dialogue between ideas and evidence may include qualitative and quantitative research components and strands. Mixed analysis has been termed both iterative and interactive, and as an “ongoing iterative exchange between the qualitative and quantitative strands that invites an engagement with the unexpected and often paradoxical” (Creamer, 2017, p. 200). It has also been referred to as a “meaningful two-way exchange of information and inferences between varied types of sources gathered and/or analytic strategies employed” (Bazeley, 2018, p. 8).

Various metaphors can be used to describe this dialogue and thereby guide mixed analysis (Bazeley and Kemp, 2012). One group of metaphors describe the mixed analysis process as a spiral (see also Bazeley, 2018), for example as a DNA double helix (Mendlinger and Cwikel, 2008; Bazeley and Kemp, 2012). Mixed methods has the potential to “spiral iteratively around the different data sets, adding depth of understanding with each cycle” (Caracelli and Greene, 1993, p. 202).

The challenge of how to engage in a spiraling analysis process can be addressed as four related questions—namely, how can a researcher create conditions that are optimal for the analysis process? How can the outcomes of the analysis process be judged? What principles can guide the analysis process itself? And finally, how can a researcher know that they are making progress during the analysis process?

The main condition for a fruitful dialogue has been described by Jennifer Greene as “engag[ing] with difference” (Greene, 2005, p. 208; Greene 2007, p. 80; see also Johnson and Schoonenboom, 2016; Johnson, 2017). Greene (2005) goes on to say that:

[A] mixed method way of thinking seeks not so much convergence as insight; the point is not a well-fitting model or curve but rather the generation of important understandings and discernments through the juxtaposition of different lenses, perspectives, and stances; in a good mixed methods study, difference is constitutive and fundamentally generative. […] In practice, mixed method educational inquiry includes multiple and diverse methods for gathering, analyzing, and representing educational phenomena within a framework that intentionally engages with the different ways of knowing and valuing that the different methods embody. (p. 208).

While creating conditions for a fruitful dialogue begins at the outset of a study, at the end of a study, the researcher faces the problem of how to judge the outcomes of the dialogue. Within the DNA metaphor, the validity criterion that is used for judging the dialogue’s outcomes is the functionality of the resultant organism:

The rigor of this type of integration derives from an inability to force any part into the organic helix; each component in the DNA sequence has a key and it has to fit in place. As in the construction of DNA, only certain sequences are possible, and only particular proteins (data) can bind together, yet variation and improvisation are important, and, as in nature, infinite variety can result. Ultimately, at the conclusion of the process, everything must “fit” and “work” so that the rigor and the validity of the integrated analysis can then be judged by the functionality of the resultant organism. (Bazeley and Kemp, 2012, p. 68).

This validity criterion can be used for judging the outcomes of mixed analysis, not for guiding the analysis itself, or for making decisions during analysis. This is because the “resultant organism” is not yet known.

Some authors have proposed that decision-making during mixed analysis should be driven by the study’s purpose. For Bazeley (2018), mixed methods integration is performed in order to meet the overall purpose of the study. Similarly, Creamer (2017) identified the purpose as what drives the decision to use mixed methods. However, although a study’s purpose may provide some direction for the researcher, it is not sufficient to guide the researcher during the analysis. This is because research purposes may change during a study. As a result, the process of research analysis may change. In addition, a study’s purpose is of little use in answering the fourth question—namely, how can a researcher know that he\she is making progress during the process of analysis? The study’s purpose does not provide feedback *on* or indicators *of* the current status of the study. The purpose does not tell a researcher how close he\she is to reaching said purpose.

This article introduces an approach to mixed analysis that intends to support researchers in making analysis decisions. It provides support by presenting information about how to assess the current status of a study and giving suggestions for the next step in the analysis. We refer to this approach as the “develop your case” approach, and we take as our starting point Bazeley’s (2018) description of data analysis within a mixed methods case study, which is based on the case study as “an empirical method that investigates a contemporary phenomenon (the ‘case’) in depth and within its real-world context” (Yin, 2018, p. 15). Bazeley claims that:

Analysis of the diverse data gathered in a case study begins with the development of a systematically organized database of information including both words and numbers. […] Writing might begin with a comprehensive case description that draws on the kinds of complementary analyses and integrative processes described already. Information is pieced together, sources compared, and documentation garnered to build a sound database of evidence that can be called on to test and support claims made regarding the case. (p. 238).

Although meant to describe the mixed methods case study, it is clear that Bazeley’s description also applies to much of mixed methods research that is outside the case study itself: many mixed methods researchers develop “systematically organized databases” of both quantitative and qualitative data. As well as this, many mixed methods researchers piece together information, compare sources, and test claims.

In this article, we present mixed analysis within the field of educational research as a process of *case development*. The development processes and their sequences help researchers determine the status of their research and provide directions for how to continue. As Ragin (1992) has pointed out, a problem associated with discussing cases is that the word *case* is used in many different ways. In this article, we refer to the *case* as a theoretical construct. Thus, when we say that a significant amount of research outside the case study aims to develop cases, we mean that the research aims to gradually refine theoretical constructs through empirical research.

This meaning of the case as a theoretical construct is illustrated by Ragin (1992):

A[n] […] investigator interested in tyranny, for example, would study many possible instances of tyranny. This investigation might lead to an identification of an important subset of instances with many common characteristics, which might be conceived, in turn, as cases of the same thing (e.g., as cases of “patrimonial praetorianism” or as cases of “modern tyranny”). Interaction between ideas and evidence results in a progressive refinement of the case conceived as a theoretical construct. (p. 9)^1^.

Throughout this article, the word *case* refers to the case as a theoretical construct. We will use *instantiation* to refer to an empirical instantiation of the theoretical construct. We will use the word *example* to refer to an empirical context that we use to illustrate or investigate the development of a case as a theoretical construct.

We define the purpose for a mixed methods study as to *acquire detailed insight into the case, subcases, and the way the subcases are moderated.* Our definition is based on Greene’s (2007) goal of “generating better understanding of social phenomenon” (p. 20). But we go beyond this by stating that, in addition, three related goals of mixed methods research are (1) to develop claims, (2) to develop what we call “subcases,” and (3) to understand how claims are moderated. These last three goals, we will show, will provide information about the status of the study and will help researchers determine their next analysis step.

Developing cases occurs at different timescales. On a larger scale, cases develop over a period of several years or even decades, involving many studies. Think, for example, about the case of the noxiousness of smoking, including the claim that smoking causes lung cancer, a claim that took several decades to fully develop. But cases also develop within a much smaller timescale, within studies. Development of cases within mixed methods studies will be the main theme of this article. We will start, however, by explaining our concepts of case development using examples of developing cases on a larger timescale.

## How Cases Develop Across Research Studies

In this section, we explain how cases can develop across several studies. We use three examples, with each one emphasizing a different process within case development.

### The Violence Example

**Figure 1:**
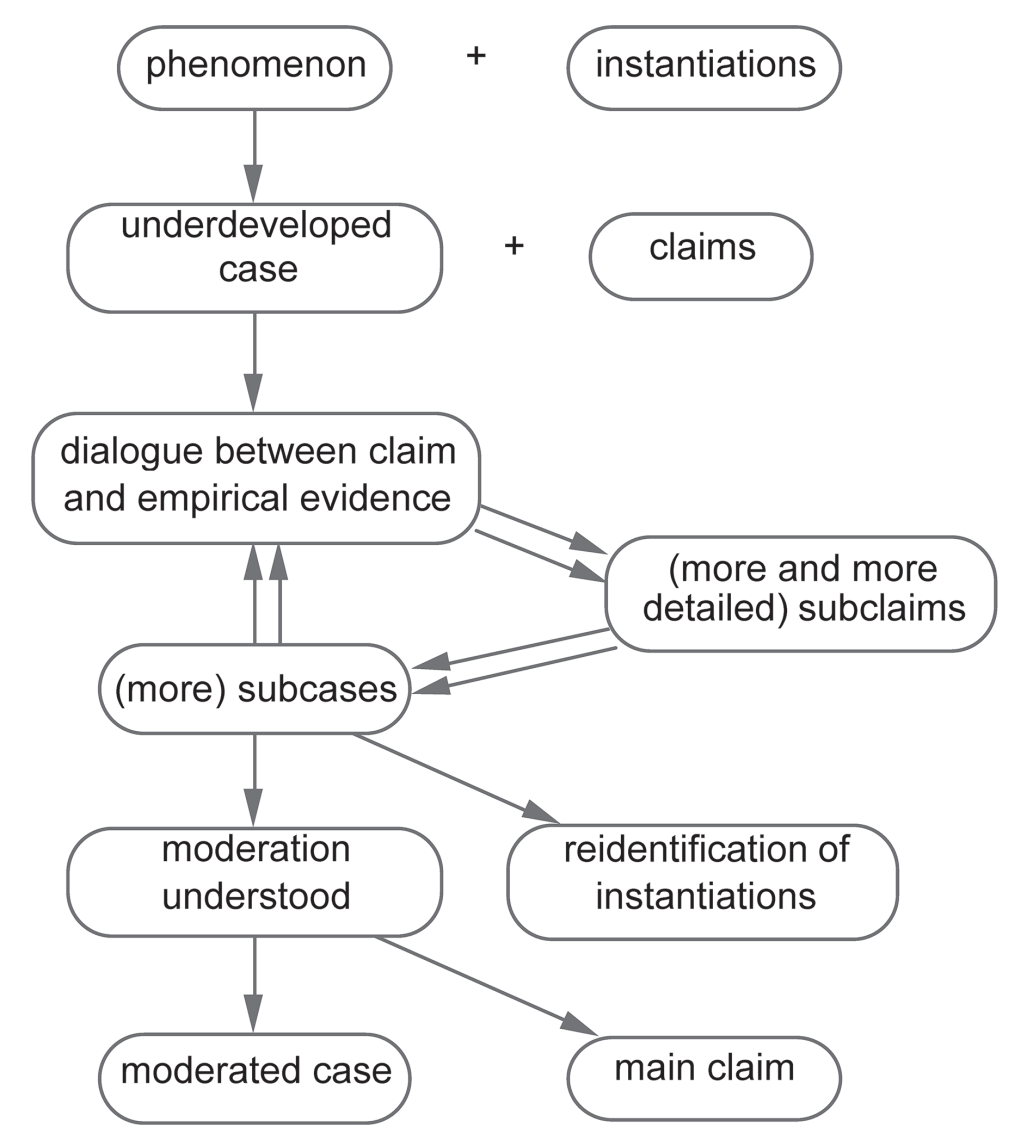
Case development across studies in the violence example: the interactive development of subcases.

Figure 1 shows the development of the case in the first example, the “violence example.”

Through a comparison of many instances of violence, Randall Collins developed the notion of violence as “a set of pathways around confrontational tension and fear” (Collins, 2009). As Collins explains:

Violent interactions are difficult because they go against the grain of normal interaction rituals. The tendency to become entrained in each other’s rhythms and emotions means that when the interaction is at cross purposes—an antagonistic interaction—people experience a pervasive feeling of tension. This is what I call confrontational tension; at higher levels of intensity, it shades over into fear. For this reason, violence is difficult to carry out, not easy. Those individuals who are good at violence are those who have found a way to circumvent confrontational tension/fear, by turning the emotional situation to their own advantage and to the disadvantage of their opponent. (p. 20).

The use of comparison helped Collins to not only develop the concept of violence, but also to distinguish and classify different types of violence, for example, the type *find a weak victim to attack* (e.g., military violence, police violence, domestic violence), and the type *fighting before an audience* (e.g., personal fights, martial arts, violence in sports). Based on his definition, Collins criticizes Bourdieu’s use of “symbolic violence” for being unrelated to other forms of violence and for not being helpful to explain the micro-sociology of violence.

Developing a case starts with identifying a *phenomenon* (Figure 1) that will be the object of research. At the beginning, this phenomenon is given a label; in the violence example, the label is “violence.” A phenomenon can be investigated empirically once researchers are able to identify specific contexts, localized in time and space, in which the phenomenon occurs. At that point, an *underdeveloped case* has come into being, and the specific contexts in which the case occurs are called *instantiations* of the case. For example, one instantiation of the case “violence” is the Battle of Waterloo, which took place in 1815.

By definition, a case has more than one instantiation. Thus, the Battle of Waterloo shares its status as an instantiation of the case “violence” with many other specific contexts, for example, with the boxing match between Muhammad Ali and Joe Frazier in New York in 1971.

Connected to each case are claims, statements about the case that are used to describe the case more precisely and to distinguish cases from non-cases. These claims are developed in an interactive cycle. Claims may refer to the case as a whole: in the violence example, to violence in general. Examples include *violence is a set of pathways around confrontational tension and fear*, *violence is difficult*, and *confrontational tension is a requirement for the coming into being of violence*.

Next to claims that apply to all instantiations of a case, claims may be developed that apply to only some of the case’s instantiations. In that case, claims are used to distinguish *subcases* (Figure 1). According to Collins’ description, the Battle of Waterloo can be considered an instantiation of the subcase “Find a weak victim to attack,” while the boxing match between Ali and Frazier is considered an instantiation of the subcase “Fighting before an audience.” The Battle of Waterloo and the boxing match between Ali and Frazier belong to different subcases of the case “violence.”

### The TRF Example

**Figure 2:**
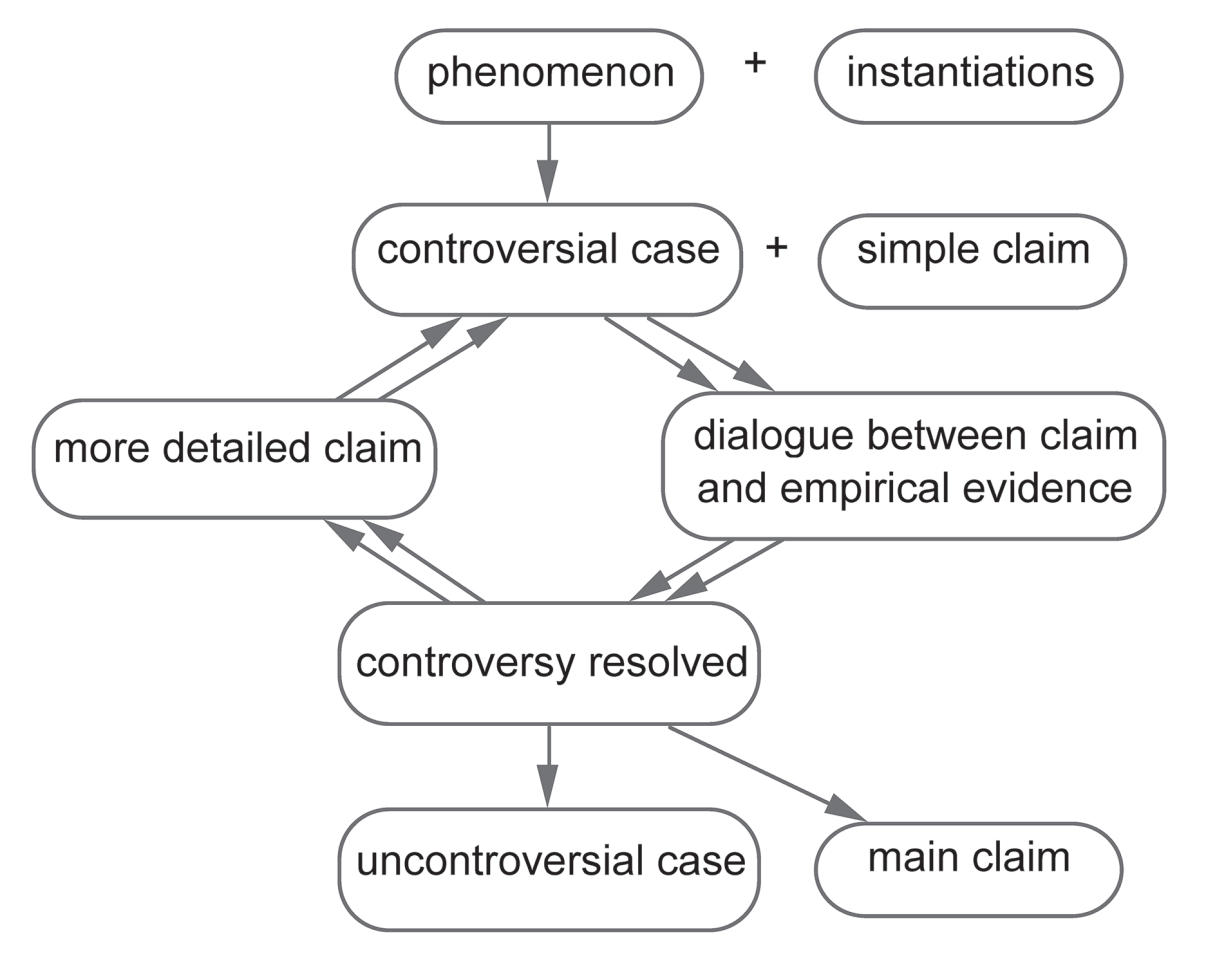
Case development across studies in the TRF example: the interactive resolution of a controversial case.

**Figure 3:**
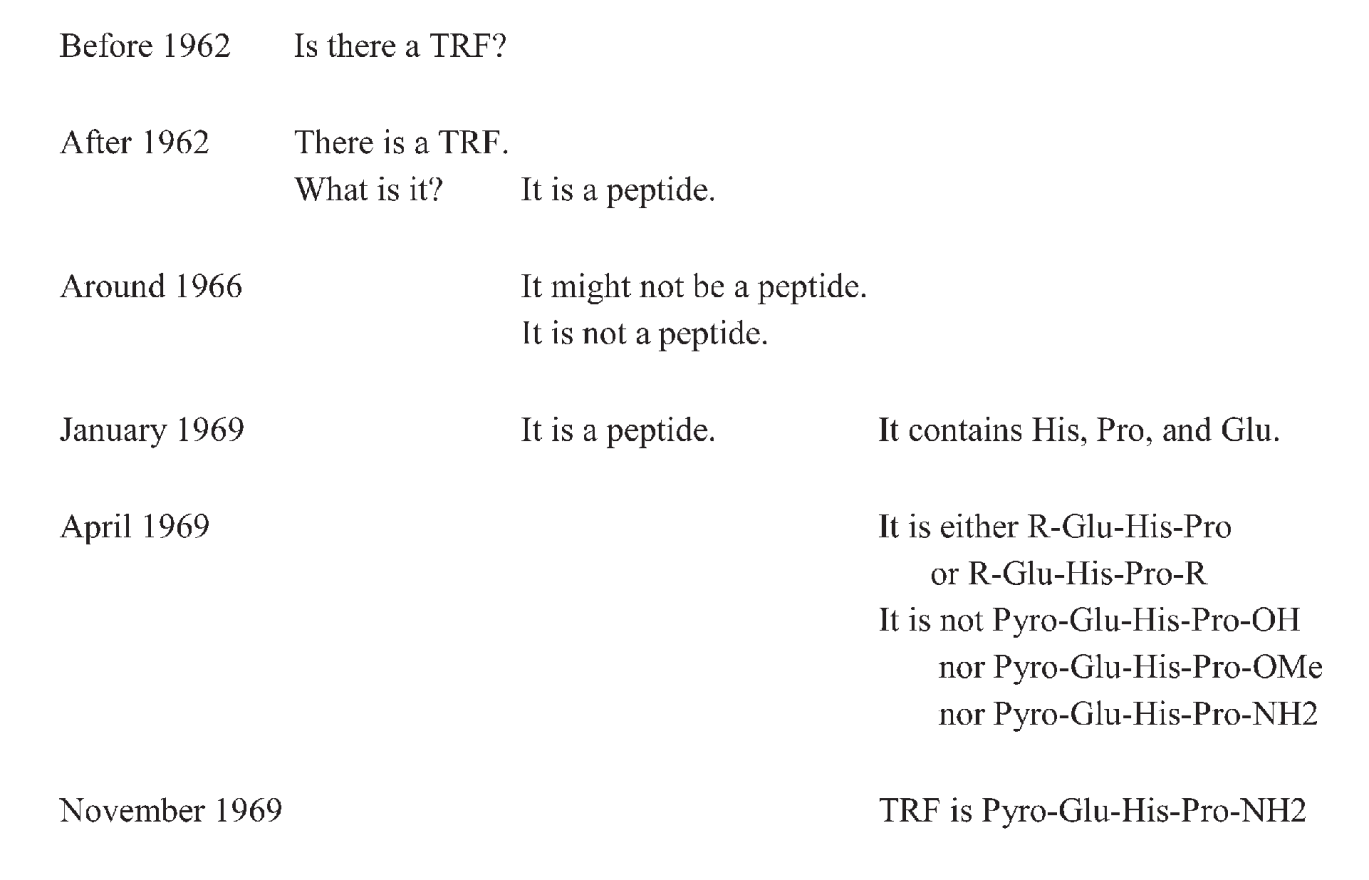
Development of TRF claims. From Latour and Woolgar, 1986. (p. 147). Copyright 1986 by Princeton University Press. Reprinted with permission, conveyed through Copyright Clearance Center, Inc.

When cases develop, they typically become *controversial* for some time: some research studies support a specific claim, while other research studies refute this claim. This is apparent in the TRF example, about the discovery of thyrotropin-releasing factor (TRF). Figure 3 shows that between 1962 and 1969, claims were developed about the existence and the chemical composition of TRF, a hormone produced by the hypothalamus (Latour and Woolgar, 1986). Figure 2 displays the structure of the TRF example.

This example starts with a phenomenon labeled “TRF.” The underdeveloped case starts with a simple claim “There is a TRF.” Before 1962, this case was controversial because it was not clear whether TRF really exists. This controversy is resolved in 1962. Researchers then proceed with a more detailed claim, “TRF is a peptide,” which is controversial for 6 years. From 1963 to January 1969, some studies support the claim “TRF is a peptide,” while other studies support the opposite claim, “TRF is not a peptide.” In January 1969, this controversy is resolved, and TRF is definitively considered a peptide. Research continues with an even more detailed claim, “TRF is Pyro-Glu-His-Pro-NH2,” which is controversial from January 1969 until November 1969. In this period, some studies support the claim “TRF is not Pyro-Glu-His-Pro-NH2,” while others support the opposite claim “TRF is Pyro-Glu-His-Pro-NH2” or still other claims. This controversy is solved in November 1969. From that moment on, TRF is a “developed case”: TRF is Pyro-Glu-His-Pro-NH2.

### The Terrorism Example

**Figure 4:**
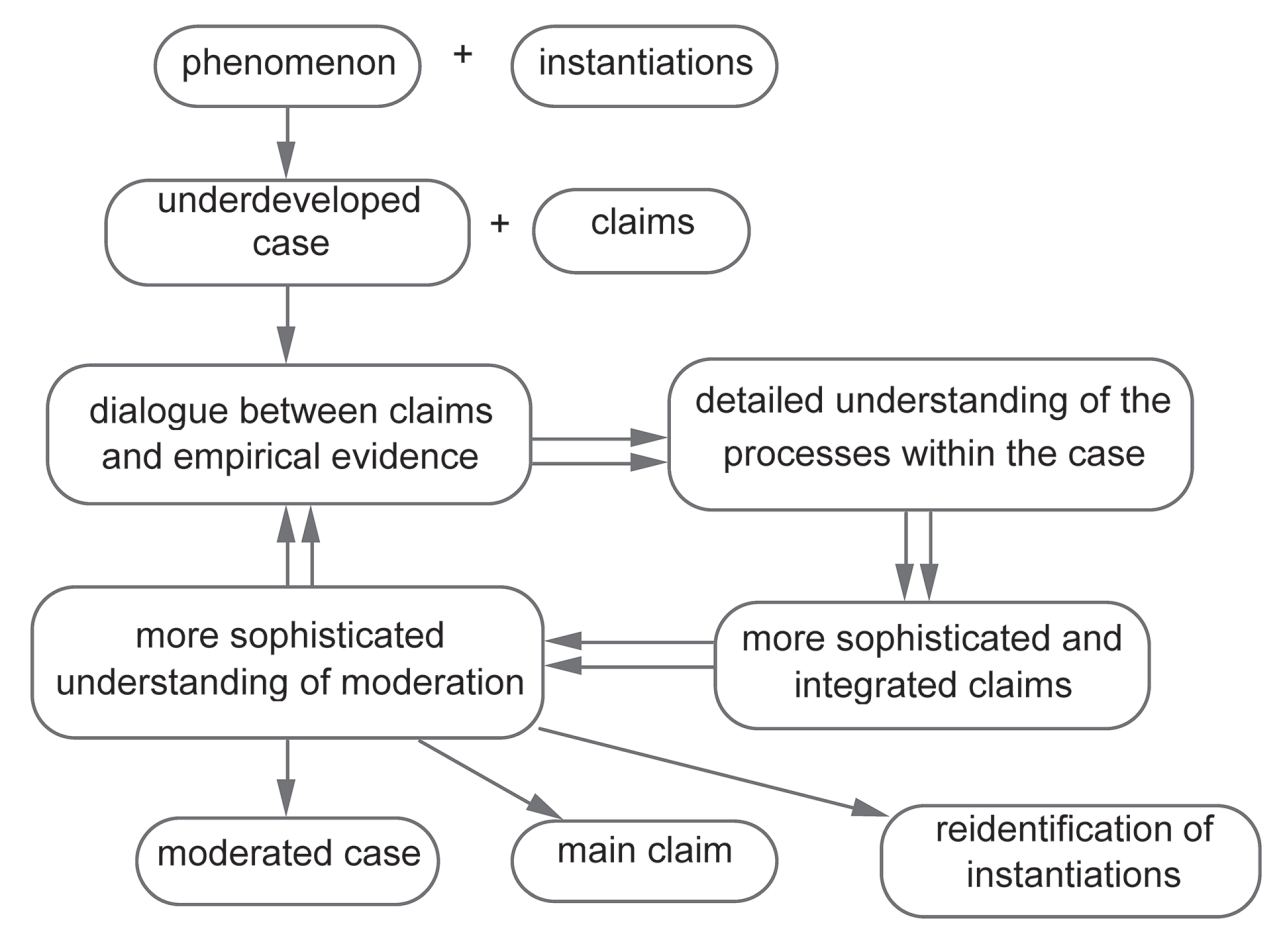
Case development across studies in the terrorism example: the interactive development of a moderated case.

The next example, the “terrorism example,” demonstrates how interacting with empirical evidence leads to a detailed understanding of the processes within the case and to the development of a *moderated case* (Figure 4).

Between 1981 and 1988, Michel Wieviorka (Wieviorka, 1992) interviewed people who were considered by themselves and the outside world as former terrorists. He interpreted and compared these instantiations to each other and to other cases of terrorism, and he developed the notion of “inversion” as a characteristic of all instantiations of terrorism. Inversion means that terrorists speak “artificially” in the name of a reference group but at the same time distort the ideas of the reference group in such a way that members of the reference group do not see themselves as being represented by the terrorists. As terrorists thus become decoupled from the group they claim to represent, their violence becomes more violent and unstoppable, as outside legitimation fails. Going back to his terrorist interviewees, Wieviorka realized that not all of them fit his developed definition of terrorism.

In the terrorism example, the researcher opens the black box of the case to understand the processes that occur in this specific context, processes that show how one event leads to the other, and that describe the processes that are essential for considering this specific context an instantiation of the case. As a detailed understanding develops, the researcher not only understands *that* a specific context is an instantiation of a particular case, but also understands in detail *how* the processes within the case take place.

The terrorism example transcends the simple claims and detailed claims of the violence example and the TRF example. It describes the processes within the case. It shows how terrorist groups become detached from the reference groups in whose name they claim to operate, and how this leads to inversion, a distortion of the ideas of the reference group. As a result, the reference group no longer considers itself represented by the terrorist group. Consequently, the bond with the reference group disappears and, through this, any control the reference group might have over the terrorist group also disappears. Due to this lack of control, the violence of the terrorist group becomes more severe and unstoppable.

Through this detailed description, the terrorism example creates *integrated claims*: two or more claims that are connected to each other. For example, the claim “terrorist groups become detached from the reference groups” and the claim “terrorist groups start to distort the ideas of the reference group” are connected: terrorist groups become detached from the reference groups, *and as a result*, they start to distort the reference group’s ideas.

Researchers use these detailed insights for four purposes: to develop additional and more detailed claims, to understand what their main claim is, to understand how their main claim is moderated, and to understand how this affects what counts as instantiations of the case. First, researchers develop various other and more detailed claims that either apply to all instantiations, and thus to the case as a whole, or to some of the instantiations, and thus to subcases. Examples of such additional claims in the terrorism example are “inversion is the result of the detachment of terrorist groups from the reference group they claim to represent” and “inversion leads to heavier and unstoppable violence.”

Second, researchers usually develop a “main” claim of the case. The main claims of the three examples, like other main claims that are the result of several years or decades of research, are claims that are not visible on first consideration of the phenomenon. They are unexpected and insightful: “violence involves pathways around resolving tension,” “TRF is Pyro-Glu-His-Pro-NH2,” “terrorism is characterized by inversion.”

Third, researchers come to understand under which circumstances their main claim does and does not apply. This is called *moderation* of their claim. In the terrorism example, inversion is such a moderator: if inversion does not occur, a terrorist group does not come into being. Similarly, in the violence example, tension moderates violence in the sense that it determines how difficult it is to be violent. As long as the tension is unresolved, violence is difficult; as soon as the tension is resolved, violence can continue for a prolonged period.

Fourth, researchers may reconsider the instantiations of a case. The development of the concept of inversion in the terrorism example showed that not all of the instantiations that had been selected beforehand were actual cases of terrorism: some did not demonstrate inversion. Similarly, in the violence example, Collins argued that Bourdieu’s cases of “symbolic violence” were not real instantiations of violence because they lack a resolution between tension and anxiety.

In summary, the three examples highlight a specific process of case development: the TRF example highlights the resolution of a controversial case; the terrorism example highlights the development of a moderated case; and the violence example highlights the development of subcases. Thus, it is clear that case development can involve various processes, and not all of these will always be present.

**Figure 5:**
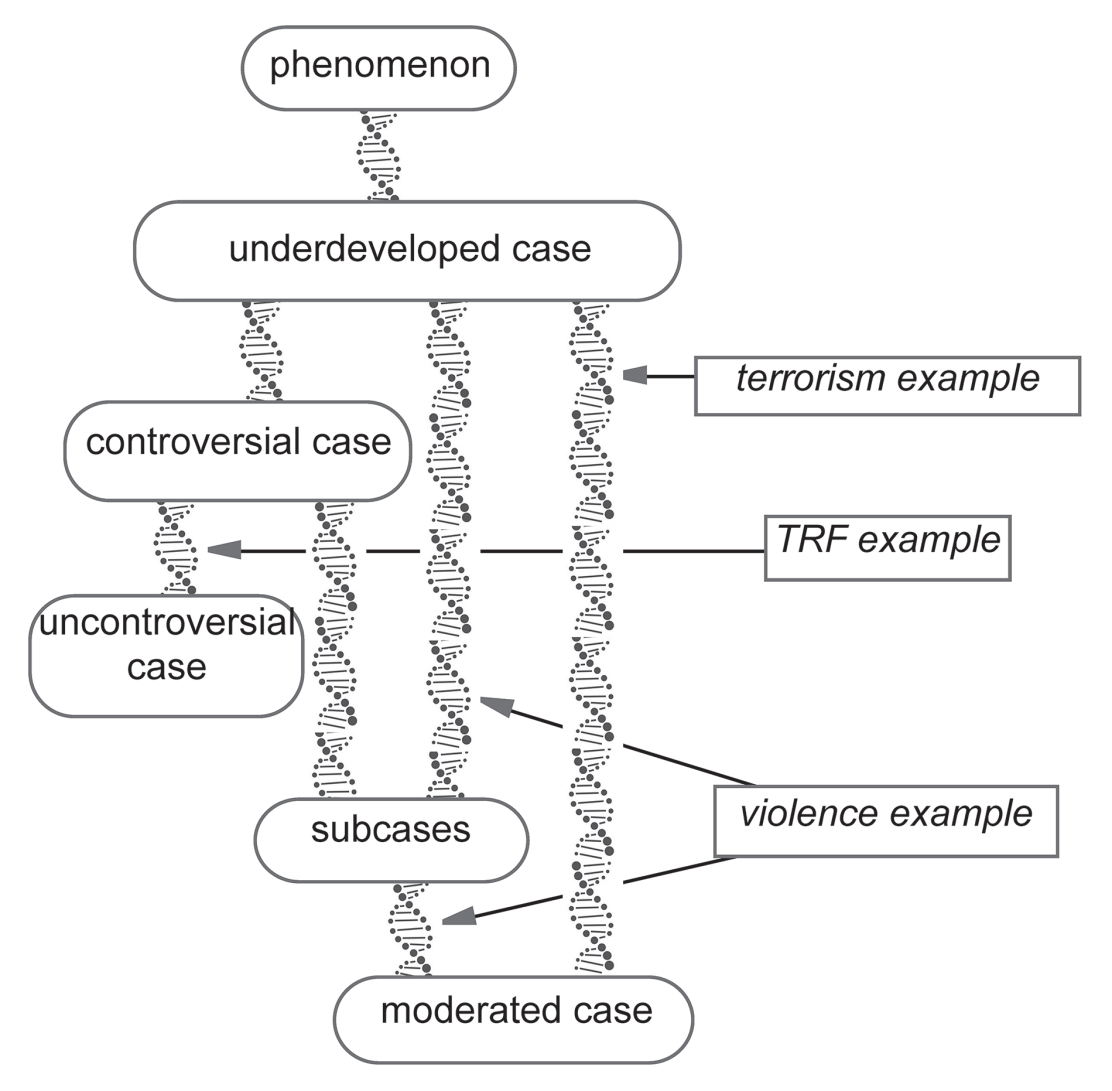
The processes of case development. The DNA double helices indicate the spiraling nature of the analysis processes.

However, the processes of controversy resolution, the development of subcases, and the development of a moderated case may and often do occur together in the development of a case. When they do, they typically occur in a specific order, as shown in Figure 5. Thus, cases often begin with a controversial case (Ragin, 1992; see the TRF example), but not always (see the violence and terrorism examples). The development of subcases comprises a typical way to resolve a controversial case (but not always; see the TRF example). The controversy is resolved by demonstrating that one claim is valid for one set of instantiations, while the opposite claim is valid for another set of instantiations. However, subcases can also develop without a preceding controversial case (see the violence example). Subcases *can* but *needn’t be* further developed by showing how they result from a moderating factor, which is present in one subcase but absent in another. Yet, a moderated case can also be developed without preceding subcases (see the terrorism example).

## Developing Cases in Mixed Methods Research

**Figure 6:**
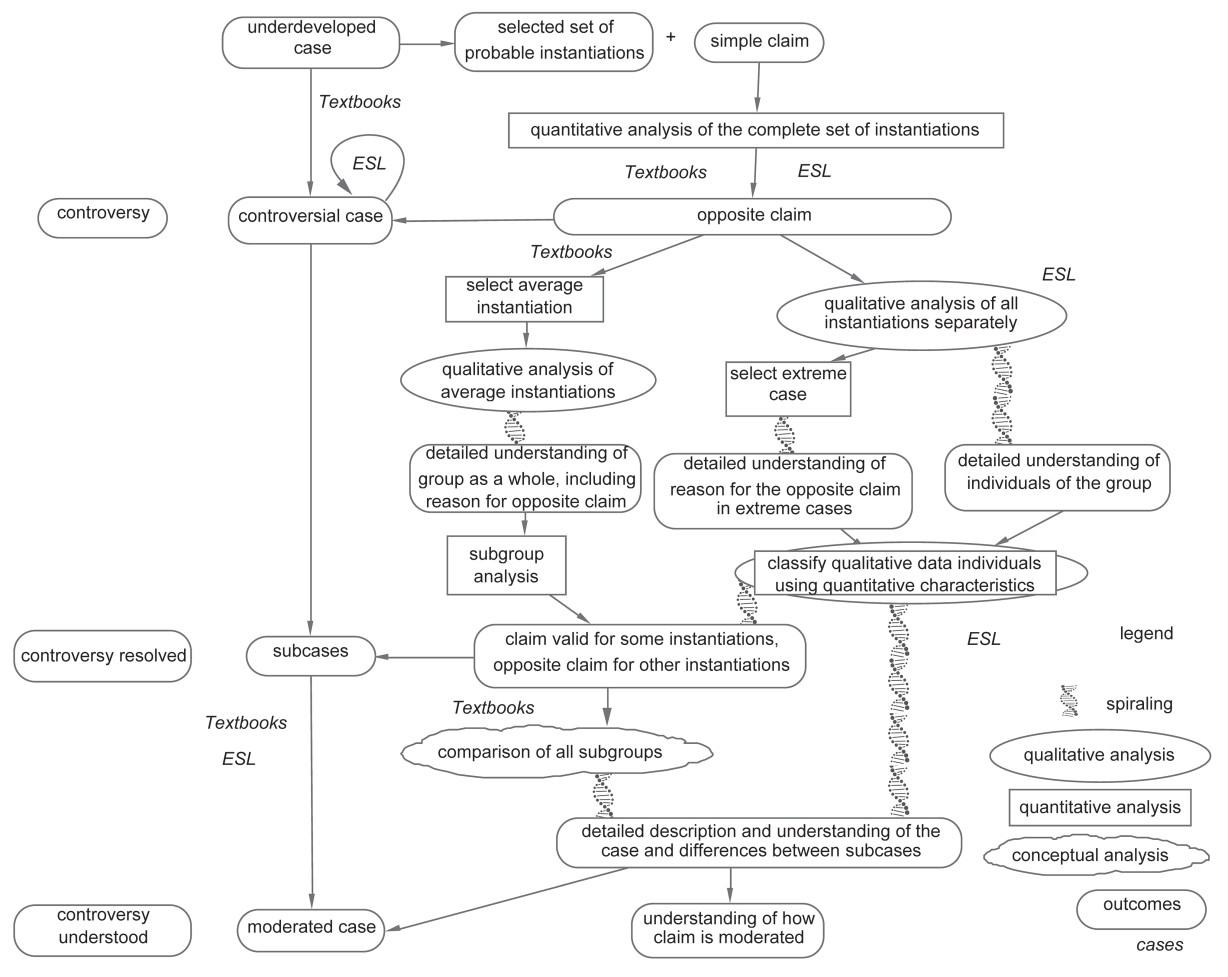
Case development in the textbooks example and the ESL example.

This case development process across studies can also be applied to case development within a study, in particular in a mixed methods study. The main tenet of our article is that we can understand mixed analysis as a process involving the resolution of a controversial case, and the development of subcases, and finally a moderated case. We will demonstrate these processes using two real-life examples of mixed methods research. The phenomena they study are, respectively, the introduction of textbooks at primary schools where textbooks had not been used before (“the textbooks example”), and language-related problems of international students who use English as a Second Language (“the ESL example”). Figure 6 shows the development of the two examples in one graphic. We can recognize the resolution of a controversial case and the development of subcases and a moderated case, described in the previous paragraph (see Figure 5).

The two examples started at a different stage of the case development process (Figures 5, 6): the textbooks example started as an underdeveloped case, while the ESL example started as a controversial case. They both next developed into a controversial case, and then into subcases, and then into a moderated case. The studies differed in their design: the textbooks example started by collecting quantitative data only, while the ESL study started by collecting both quantitative and qualitative data.

Figure 6 presents the various processes in relation to two real-life examples. It shows the data analyses performed, with the qualitative analyses in oval boxes and the quantitative analyses in rectangular boxes. Outcomes are shown in rectangular boxes with rounded edges. Qualitative and mixed analyses that involve a spiraling analysis process are indicated by the DNA double helix that connects the description of the analysis with its outcomes. We will now describe the two examples separately, beginning with the textbooks example.

## The Textbooks Example

### From Underdeveloped Case to Controversial Case

As said before, cases typically become controversial for some time: some research studies support a specific claim, while other research studies refute this claim. Individual studies can make a case controversial when they show that a claim previously assumed to be valid does not apply to the instantiations of the study. Showing that a claim does not apply is typically accomplished through quantitative research. Such claim testing was done at the start of both examples. This test was performed using quantitative data analysis, indicated in Figure 6 as a rectangular box. The quantitative test at the beginning of the textbooks example turned an underdeveloped case into a controversial case:

In an intervention study by Glewwe et al. (2009), 100 primary schools were randomized to treatment conditions. Unlike previous studies, Glewwe et al. (2009) found that the provision of textbooks to primary school children in rural Kenya had no effect on students’ quantitative test scores. (Schoonenboom et al., 2018, p. 274).

As a result of the quantitative test, the case of the textbooks example became controversial: contrary to other sites, where textbooks had an effect on students’ quantitative test scores (claim), in rural Kenya, textbooks did not have an effect on students’ quantitative test scores (opposite claim). This was achieved through a test that fit the textbooks example as a quasi-experiment. The test measured the influence of one dichotomous independent variable, the intervention, on a continuous dependent variable, the quantitative test scores.

### From Controversial Cases to Subcases

After the quantitative test, the case of the textbooks example had become controversial. This controversial case was subsequently developed into subcases (Figures 1, 6). This means that the researcher tries to solve the controversy by showing that the claim applies to a subset of the instantiations and does not apply to another subset. The textbooks example proceeded as follows:

From the quantitative test score list, the researchers selected for each of 50 schools one child with a median score. They went to each school and asked the selected child who scored at the median to read a fragment from his or her textbook and answer a few questions about what was read. This revealed that in the lower grades, most median children, up to 85% in Grade 3, were unable to read their textbook. One of the problems with reading turned out to be that these difficult textbooks were written in English, which was not the children’s native language. Not surprisingly, most median children were unable to answer questions about the textbook’s contents, thereby supporting the “no effect” derived from the lack of a significant correlation in the quantitative test score analysis. Further subgroup analysis of the quantitative test scores showed that providing textbooks did have an effect on the test scores of high achievers, who were able to read their textbooks (Schoonenboom et al., 2018, p. 274).

The process from controversial case into subcases did not proceed directly. Qualitative analysis was performed to reveal the reason behind the lack of an effect. This knowledge was subsequently used to develop the subcases. First, the researchers obtained a detailed understanding of the reading processes of the children for whom the textbooks failed to have an effect. As they wanted to study in depth the process on average, they decided to observe in depth the “average” child. Thus, they started by using their quantitative data to select a child with a median test score in each class (“select average instantiation” in Figure 6). Next, the researchers observed the average children (“qualitative analysis of average instantiations” in Figure 6). It became clear that these children were unable to read their textbooks, which were in English, not their native language. Thus, the researchers obtained a detailed understanding of the processes involved and were able to explain why textbooks did not have an effect.

This did not turn the controversial case into subcases yet. For this next stage, the researchers went back to their quantitative data and conducted a subgroup analysis, in which they focused on high achievers, and compared the effect of textbooks on high achievers. This led to the development of two different claims for two different groups: textbooks had an effect for high achievers but not for the other students. Thus, out of the controversial case, the researchers were able to construct two subcases, one case for high achievers, for whom providing textbooks had an effect on their quantitative scores, and one case for the other students, for whom providing textbooks did not have an effect. In this way, they resolved the controversial case. The case was no longer controversial because there was no longer a controversy about whether providing textbooks did or did not have an effect. Instead, they now had an effect for only one specific group: high achievers.

### From Subcases to a Moderated Case

After establishing the subcases, the researchers tried to find an explanation for why the textbooks had an effect for high achievers. They tried to identify a factor that was present in the high achievers and lacking in the other students. Such a factor is called a moderator (Hayes, 2017). The factor moderates the effect of providing textbooks on quantitative test scores. They identified this factor through reasoning: if the inability to read the textbooks prevents the average child from learning from them, the high achievers are most probably able to read their textbooks. Thus, while the ability to read the textbook earlier is part of the explanation of the controversial case (the average child lacks this ability), it later becomes the moderating factor, as it explains the existence of the subcases: textbooks have an effect only for those children who can read them.

## The ESL Example

### Contributing to the Controversial Cases

Different from the textbooks example, the ESL example started as a controversial case, and the ESL example contributed to the controversy:

Lee and Greene’s (2007) purpose was to understand the predictive value of scores on an English as a second language (ESL) test—the Computerized Enhanced ESL Placement Test (CEEPT)—for international graduate students at a large public university in the United States in relation to their academic performance —measured as Grade Point Average (GPA)—and their language difficulties in courses during their first semester. The results of previous studies had been inconclusive. Some studies showed a less than perfect command of the English language had a negative effect on grades, while this effect was lacking in other studies. Lee and Greene correlated the scores of the 100 students who had taken the CEEPT test with their GPAs, which resulted in a non-significant correlation.

The ESL example started as a controversial case. Some studies had shown that language problems had an effect on international students’ first semester GPA (claim), while other studies had not demonstrated an effect (opposite claim). As in the textbooks example, the ESL example started with a quantitative test of the claim, which showed a lack of a significant correlation between language abilities and first semester GPA. Thereby, the ESL example contributed to the controversy by providing yet another example in which the opposite claim (no effect) held. Different from the textbooks example, the ESL example was a correlational study, and its variables were continuous variables that were not experimentally manipulated. Data analysis involved establishing a correlation between the continuous independent variable score on the CEEPT test and the continuous dependent variable, GPA.

### From Controversial Case to Subcases

Like the textbooks example, development of the ESL controversial case into subcases started by performing a qualitative data analysis to try to find an explanation for the lack of an effect of CEEPT scores on first semester GPAs:

100 students had performed the ESL-test, 55 students had done a self-assessment, and 34 faculty members had evaluated their students. Interviews had been held with 20 students and 10 faculty members. “Extreme case analysis showed that students’ views of academic success and their perceived levels of background knowledge can help explain low GPAs relative to the CEEPT scores.” (Lee and Greene, 2007).

In the ESL example, extreme instantiations, rather than the average instantiation, were selected to explain the nonoccurrence of an effect of language problems on GPA. The extreme instantiations were individuals to whom the effect did not apply, that is, students with a high CEEPT score and nevertheless a low GPA. Next, Lee and Greene (2007) connected their qualitative data to their quantitative data:

And a display that connected quantitative CEEPT scores to qualitative interview quotations in a table revealed that the CEEPT scores differentially predicted perceptions of academic performance for both students and faculty members. (p. 380).

Combining the quantitative and qualitative data showed that the connection between CEEPT score and GPA was different for different instantiations (individuals).

At this point, Lee and Greene (2007) had not developed subcases yet, but had shown why effects were lacking at the individual level. In a reanalysis, we took Lee and Greene’s published data one step further by developing real subcases. In our analysis, we used the quotes that had been published in Lee and Greene (2007) concerning what both successful and unsuccessful students had said about their language problems and how this influenced their GPA.

We constructed a sortable table, in which the statements of students were put next to their language test score and first semester GPA. By sorting and resorting the three columns of this table, we discovered an interesting pattern. We identified four successful students with the highest GPA of 4 in spite of a CEEPT score of less than 4. Three of the four successful students mentioned some method of compensating for their language problems. One student compensated for his or her misunderstanding of the professor by reading the textbook, another other compensated for problems speaking English by calling on a strong mathematical background, and yet another compensated by careful reading. A comparison with their less successful counterparts showed that the compensation strategies mentioned by three of the four successful students were not mentioned by any of the unsuccessful students. This finding could explain the lack of an effect of CEEPT on GPA: good students have strategies to compensate for their language problems.

We developed this subcase by creating a table in which the qualitative and quantitative data of each individual were shown next to each other. This is displayed in Figure 6 as an analysis that involves both qualitative and quantitative data (a rectangular box within an oval box). By coding the qualitative utterances and sorting the table on the quantitative scores, we saw that one group with specific quantitative characteristics (a less than perfect CEEPT in combination with a perfect GPA) was characterized by successful strategies for overcoming their language problems. Thus, we were able to distinguish three subcases: students with a less than perfect CEEPT and a perfect GPA, students with a less than perfect CEEPT and a less than perfect GPA, and students with a perfect CEEPT.

### From Subcases to a Moderated Case

The three subcases quite naturally led to the identification of the moderator: successful strategies for overcoming language problems. These were mentioned by the students with a less than perfect CEEPT and a perfect GPA, but not by students with a less than perfect CEEPT and a less than perfect GPA. The successful strategies for overcoming language problems prevent the effect of a less than perfect CEEPT on GPA from occurring.

## Similarities and Differences Between the Real-Life Examples

**Figure 7:**
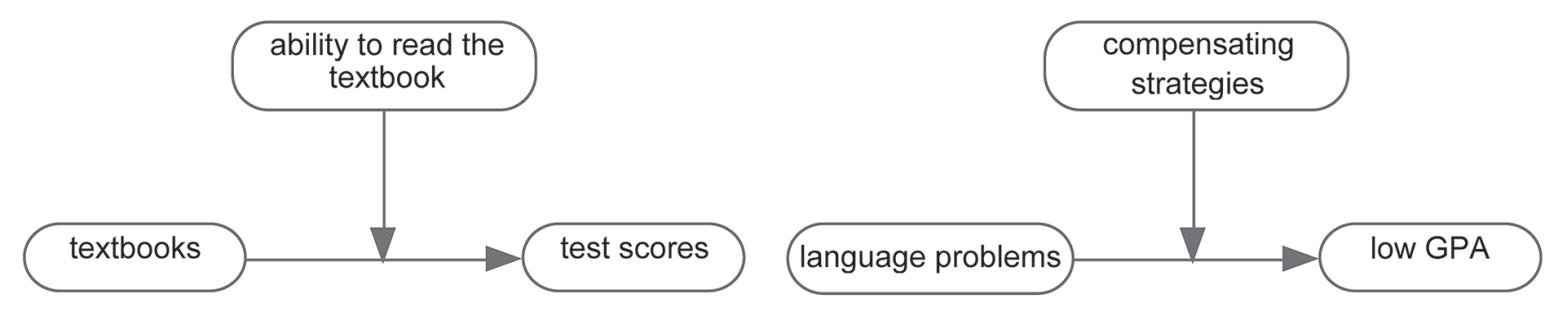
The moderated claims at the end of the study in the textbooks example and the ESL example.

We have provided two real-life examples of mixed methods educational research in which a case is developed within one study. Both examples showed development from a controversial case into subcases into a moderated case. And in both examples, both qualitative and quantitative research played more than one role, and these roles were the same for both examples. In both examples, quantitative data analysis was used at the beginning to test a simple claim, which turned the cases into controversial cases. Also in both examples, the same quantitative data were used to select the cases for the qualitative data analysis that was to provide an explanation for the lack of an effect. Finally, in both examples, the same quantitative data were used in identifying the subcases. In both examples, qualitative data analysis was used to obtain detailed insight into the processes of the case. This qualitative data analysis fulfilled two important functions. First, it explained the lack of an effect derived from the quantitative research. Second, it was used to understand moderation, that is, to understand which factor was responsible for the differences between the subcases (Figure 7).

The examples differed in the data analysis methods that were used. Data analysis methods were adapted to the specific characteristics of each study. First, the type of quantitative data analysis was different depending on the research design. In the textbooks example, a quasi-experiment, a regression analysis of a nominal variable, the intervention, was used to establish the lack of an effect. In the ESL example, a correlational study, a correlation between CEEPT and GPA was calculated to test the existence of an effect.

The examples also differed in their strategy for sampling instantiations that were used for finding an explanation for the nonoccurrence of the effect. This difference originated in differences between a quasi-experiment (textbooks example) and a correlational study (ESL example). In the textbooks intervention, the researchers tried to find an explanation for the nonoccurrence of an overall effect by investigating the average child as a representative of the experimental group as a whole. In the ESL example, where the independent variable was continuous, the researchers focused on individuals at both ends of the spectrum for whom the effect was not there: individuals with a low CEEPT score and a high GPA, and individuals with a high CEEPT score and a low GPA.

## Discussion

### Characteristics of the Case Development Approach

The mixed analysis processes described in this article have five characteristics. First, and most importantly, they depend on the status of a case as either a controversial case, a collection of subcases, or a moderated case. In addition to this, mixed analysis processes depend on the fact that, by definition, a case has more than one instantiation; they also enable and make use of rich databases; their claims are always tentative; and their final conclusions may differ in strength. We discuss these last four characteristics first.

The idea that the phenomenon in a case is not unique but that there are always various instantiations of a case distinguishes case development from some forms of qualitative research. If one assumes that a phenomenon is unique, one can acquire deep insights into the processes involved through qualitative research, meaning that there is no need for quantitative research. If, conversely, one assumes that a case is not unique but that there are various instantiations of it, then quantitative research can fulfill some useful roles. For example, it then makes sense, at the beginning of the study, to make a preliminary distinction between instantiations and non-instantiations and to select a set of instantiations for investigation. For this purpose, quantitative data are very useful. Similarly, quantitative data can be used to make a preliminary distinction between various subcases.

Second, data collected for case development are typically used more than once and for different purposes. In both examples, quantitative data led to the identification of controversial cases and/or subcases, while qualitative data led to a detailed understanding, and combining the two led to the identification of moderation. The database was not only used once, for testing a claim, but was also used for sampling and in developing subcases and determining moderated factors.

A third characteristic of our approach is the tentativeness of the claims, the case, and its instantiations. This became most clear with the controversial cases. The two controversial cases were only temporarily controversial. Both were developed into subcases during the study. In the end, the textbooks example consisted of two subcases: one group of instantiations to which the effect did not apply and another group of instantiations to which the effect did apply. The ESL example did not end with one group of instantiations for which language problems did not affect GPA. Instead, for one specific group—students with compensation strategies—the effect did not apply, while for another group of students with language problems, the effect did apply.

As a result, the relevance of the claim of the controversial case to the whole set of instantiations is temporary as well. At the end of the study, it is no longer important to know whether textbooks have an overall effect for the whole set of instantiations, or to know whether language problems overall have an effect on GPA. Once the researcher has described and understood the moderating factors, the overall effect loses its meaning.

Fourth, there is one remarkable difference between the two examples at the end of the analysis process: the status of the moderated claim or, more precisely, the strength of the evidence for the moderated claim. The evidence in the textbooks example was strong. The qualitative research in the textbooks example was targeted at one possible moderator: the ability to read the textbooks. Detailed information was acquired regarding the question of whether the average child could read and understand a textbook. Yet, the evidence concerning why there was an effect for high achievers remains implicit: it was assumed, rather than tested, that high achievers are able to read their textbooks. The evidence in the ESL example is very weak. It is only a first conjecture. Nevertheless, it is an interesting conjecture that is worth further investigation.

The extent to which the claims are generalizable is different as well. None of them can be generalized statistically, as none of them involves a randomly drawn sample. The claim in the textbooks example is a very strong candidate for theoretical generalization: it is very likely that being able to read a textbook is a requirement for being able to understand the contents of the textbooks, and that this is a requirement for textbooks to have an influence on students’ grades. Finally, as said above, the claim in the ESL example is a conjecture. It is neither generalizable nor can it be said to be valid for the sample as a whole.

### How Knowledge of a Case’s Status Can Guide Mixed Analysis

In this article, we presented mixed methods analysis as a process of case development, which typically proceeds by resolving a controversial case and developing subcases and a moderated case. We are now able to distinguish two different ways in which these processes of case development can guide mixed methods researchers through the process of mixed analysis.

First, knowing whether the case is currently an underdeveloped case, a controversial case, or whether it contains subcases or is a moderated case can help the researcher decide which analysis to perform next. When a case is underdeveloped or controversial, the researcher can decide to use quantitative data to test whether the claim applies to the groups of instantiations that they investigate. This group analysis may prove that the claim does not apply, and this either confirms that the case is controversial (ESL example) or creates a controversial case (textbooks example). When the case has already been developed into subcases, the researcher can decide to use qualitative data to discover and develop the moderating factors that distinguish said subcases.

Second, knowing whether one is working toward resolving a controversial case, toward developing subcases, or toward developing a moderated case can also help a researcher to decide which analysis to perform next. If a researcher is working toward the development of subcases, it makes sense to return to the quantitative data to see whether there is a differential effect for different groups.

If a researcher is working toward moderated claims, it makes sense to use qualitative data analysis to uncover the processes in the case and possible moderating factors of the effect. This applies in at least two stages of the analysis process: first, after the quantitative analysis at the beginning, when researchers may use qualitative data to obtain an explanation for the group as a whole. The factors that constitute these explanations are typically those factors that may prove to be moderators later on. Second, after establishing subcases, a researcher may use qualitative data analysis to see whether the processes for the subcases are different, in which case they have discovered moderating factors.

**Figure 8:**
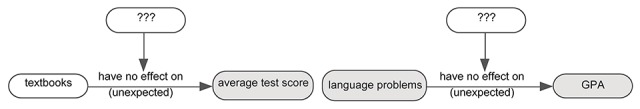
Sampling in the textbooks example and the ESL example. Variables involved in sampling are represented by light grey boxes.

Knowing that one is working toward the development of a moderated case can also be used by the researcher to determine the sampling strategy. Figure 8 shows the moderated effect in the two studies, with the as yet unidentified moderated factors as question marks.

In the textbooks example, the researchers knew after the first quantitative test that textbooks did not have an effect on the quantitative test scores on average. Therefore, it made sense to select individuals representing the “average child” to investigate what moderating factor caused the nonoccurrence of the effect in this group. In the ESL example, the researchers knew after the first quantitative test that language problems unexpectedly proved not to affect GPA. Therefore, it made sense to investigate various combinations of CEEPT scores and GPA, to see under which circumstances language problems lead to a lower than expected GPA.

In summary, we have demonstrated that knowledge of a case’s status can guide mixed methods research in various ways. But the general purpose that we have formulated for mixed methods research acts as a steering guide as well. In the introduction, we formulated the general purpose of mixed methods research as *to acquire detailed insight into the case, the subcases, and the way the subcases are moderated.* In both examples, researchers did achieve insight into their case, their subcases, and the moderated case. Thus, the outcomes of both examples are remarkably similar, in spite of considerable differences between the examples at the beginning. Our general purpose in mixed methods research puts the case, the subcases, and the moderated case in a specific order and can thus be used as a basis for what to do next. The researchers of both examples used this order. When their case was controversial, the researchers next resolved the controversy by developing subcases; and when they had developed subcases, the researchers went on to understand the moderators that led to the subcases.

### Relation to Other Approaches

Several approaches to mixed methods research state that qualitative and quantitative research provides different perspectives on a phenomenon. These different perspectives were called, for example, “variance theory” versus “process theory” by Maxwell (2013) and Mohr (1995). Variance theory, according to Maxwell et al. (2015), is connected to quantitative research, in which causality is studied on the basis of connections between variables. Process theory, aimed at describing events and processes that connect these events, is “fundamentally different from variance theory as a way of thinking about scientific explanation” (Maxwell et al., 2015, p. 227). Different perspectives are also a characteristic of Greene (2015), who described an example of a mixed methods evaluation as a combination of a postpositivist strand, using a quasi-experiment, and a constructivist strand, using mini-case studies.

Our approach is different. We do not consider qualitative and quantitative research as providing different perspectives but rather as playing two different roles within one overall perspective. While Maxwell (2013) and Greene (2015) related qualitative research to studying cases, *we view both qualitative and quantitative research as playing different roles within a case development approach*. For Maxwell and Greene, the question remains how to combine the different perspectives, how these different perspectives actually work together in a study (Mol, 2002). This question is answered in our approach, by stating that qualitative and quantitative research work together in developing cases and their claims. In this development process, we view qualitative data as necessary for obtaining detailed insight into the processes within the case and for understanding moderation, and quantitative data as necessary for identifying possible instantiations of the case, for testing claims, and for distinguishing subcases.

The case development approach includes an approach to sampling, which is similar to Emmel (2013). Handbooks on qualitative and mixed methods research provide many different methods for sampling individuals (among others Patton, 2014). In our approach, the choice of the sample depends on the status of the claims and the moderation that the researcher is investigating (see Figure 8): researchers should sample their individuals on the basis of their values on the dependent variable related to the claim (quantitative test scores in the textbooks example and GPA in the ESL example), and, if relevant, on the basis of their values on the independent variable related to the claim (CEEPT score in the ESL example).

Our approach to research as case development has consequences for our view on triangulation. Traditionally, triangulation is considered an attempt to support or reject a specific claim. This form of triangulation is found at the beginning of our cases, where testing a claim led to a controversial case: the claim was not confirmed. In our approach, however, *the outcome of testing a claim only has temporary relevance*. Testing claims has to be followed by attempts to resolve controversial cases by developing subcases and by understanding the moderating factors. Triangulation is not the aim or the end result of a mixed methods study, it is only the beginning. The aim of mixed methods research is detailed insight into the case, including the start claim and/or more differentiated claims, and how they are moderated.

Our approach is different from a grounded theory approach. Both within a study and over time, the process of case development has two characteristics: first, it develops through a stage of being a controversial case, in which some studies support one claim, while other studies support the opposite claim. This stage of a controversial case was visible within our two cases. A second characteristic of our approach is that claims become more detailed over time and that they change (think of the TRF example). Thus, the theory does not gradually emerge from the data but instead goes in many and sometimes conflicting directions. Any new study may lead to new insights and further developments. That means that we do not work according to the principle of theoretical saturation (Emmel, 2013; Becker, 2014).

We agree with many mixed methods scholars that design is very important. In our approach, it is important that researchers think very carefully about the quantitative and qualitative data they collect. One reason is because researchers go back to their collected data several times. At the beginning, researchers will use their quantitative data for testing an effect. Later on, they will use the same data to select a sample for qualitative research. Still later, they will return to their quantitative data to perform a subgroup analysis. Similarly, they will first use their qualitative data to explain the effect or lack of an effect derived from their quantitative research, but later they will use the qualitative data to describe the processes of the case and to investigate moderation.

The stages and type of analyses used for our case development process do not depend on the order in which the quantitative and qualitative data are collected. Our examples differed in their design: the ESL example started as a concurrent design (Creswell and Plano Clark, 2018), in which quantitative and qualitative data were collected at the same time. The textbooks example started as an explanatory sequential design with the collection of quantitative data only, later followed by qualitative data collection. Yet the case development process and the type of data and analysis used were the same: first developing a controversial case by testing a claim using quantitative data, then proceeding toward subcases by first trying to explain the lack of an effect through analysis of qualitative data and using the quantitative data to select a sample for this qualitative data collection or selection. Then, the qualitative data in combination with the qualitative data are used to establish subcases. Finally, in both example cases, the researchers tried to understand the moderating factors.

We have presented our approach as if it were a general approach to mixed methods research. Of course, we cannot prove the usability of our approach as a general approach on the basis of just two examples, but we have reasons to assume that the approach might provide a good basis for mixed methods in general. First, the approach proved able to describe two mixed methods studies that at first sight appeared to be quite different, one being an experiment, the other a correlational study, and one being a sequential design, the other a concurrent design. We have applied the approach successfully to two additional different examples, which we cannot include here due to lack of space.

Second, we saw that we can use our approach both for describing mixed methods studies and for the development of research more generally, across research studies. This adds to the credibility of our approach. Actually, the ESL example is partly a case across studies because we developed the subcases ourselves on the basis of an existing study by Lee and Greene (2007). At a minimum, we can say that our approach is applicable to a range of studies and particularly to mixed methods studies.

## Author Contributions

JS performed the research and wrote the article.

### Conflict of Interest Statement

The author declares that the research was conducted in the absence of any commercial or financial relationships that could be construed as a potential conflict of interest.

## Footnotes

^1^While this fragment clearly illustrates the meaning of *case* as a theoretical construct, it also unwittingly demonstrates how difficult it is to separate the various meanings of *case*. Thus, Ragin begins by distinguishing “[empirical] instances of a case [as a theoretical construct],” identical to our “instantiation of a case.” In the second sentence, however, Ragin speaks of “cases of the same thing.” In this sentence, *cases* does not refer to the case as a theoretical construct, but to the case as an empirical instance. Thus, to avoid confusion, it would have been better if Ragin had said “as instances of the same subcase (e.g., as instances of the subcase ‘patrimonial praetorianism’ or as instances of the subcase ‘modern tyranny’).”
